# Factors Affecting the Public Acceptance of Extramarital Sex in China

**DOI:** 10.3390/ijerph18115767

**Published:** 2021-05-27

**Authors:** Nian Liu, Zekai Lu, Ying Xie

**Affiliations:** Department of Sociology, School of Public Administration, Guangzhou University, Guangzhou 510000, China; liunian@gzhu.edu.cn (N.L.); 1903500028@e.gzhu.edu.cn (Z.L.)

**Keywords:** extramarital sex, public acceptance, influencing factors, zero inflation

## Abstract

There is a lack of quantitative studies on the acceptance of extramarital sex in China. Based on data from the Chinese General Social Survey 2013 (CGSS2013), this paper used a zero-inflated Poisson regression model to analyze the factors influencing the public’s attitudes toward extramarital sex. When other variables were controlled, groups of younger ages, higher educational levels, and stronger tendencies toward “liberalization” and non-Islamic beliefs were more tolerant toward extramarital sex, whereas gender and Christian beliefs had no significant influence. In this regard, family and marriage counseling, and society’s moral tolerance and social control of religion are discussed, and further research on cross-cultural verification is needed.

## 1. Introduction

Extramarital sex refers to a sexual relationship between a married person and another person (married or unmarried) outside of marriage. Extramarital sex is usually accompanied by extramarital affairs, but the conceptual connotations of the two are not exactly the same. Extramarital sex can occur with extramarital affairs, or it may be a “one-night stand” or even simple sexual behaviors and other behaviors that have little to do with affection [1]. Analysis of the acceptance of extramarital sex is an important topic in sociological and psychological studies. Most studies are based on data from Western developed countries. These studies take sociodemographic characteristics as the independent variable and the acceptance of extramarital sex, same-sex marriage, and homosexual groups as the dependent variables to analyze the relations between the variables [2,3]. For example, the US General Social Survey has included issues of extramarital affairs and extramarital sex for many years, providing evidence for empirical research. Studies from the database show that society’s tolerance for extramarital sex is on the rise [4].

Among the factors that influence the public acceptance of extramarital sex, the key study variables are religion, education, gender, age, and social freedom. Higher levels of education and lower ages often have a positive impact on the public acceptance of more open sexual behavior [5]. Given the importance of religion in Western countries, many studies indicate that religious (Christian) beliefs have a significant negative influence on the tolerance toward extramarital sex [6]. Some detailed studies explore the differences in the acceptance of sexual behavior between different religious sects (such as different groups within Christianity) and between different races [7].

Different dynasties in ancient China had much different views on extramarital sex. For example, in the Tang Dynasty, people regarded extramarital sex more as a fashion; after the Song and Ming Dynasties, extramarital sex was regarded as heretical and thus prohibited. In general, Chinese society takes a cautious and conservative attitude toward sexual behavior. In contemporary times, extramarital sex is regarded as immoral at the moral level and deviant at the social ethical level; although the Marriage Law does not qualify “extramarital sex” at the legal level, it has established “husband and wife shall be loyal to each other and help each other” as a basic principle, and in legal practice, there is a tendency to protect the legal rights and interests of the spouse as a victim of extramarital sex. Contemporary Chinese society is undergoing a period of intense social transformation. Rapid urbanization has enabled an increasing number of rural people to enter the strange urban society from the former rural acquaintance community, and the effect of the original community norms for the control of individuals has been greatly weakened; meanwhile, with the increase in population mobility, the disintegration of the urban traditional unit system, and the increase in individual anonymity, when a person commits a deviant act of “extramarital sex,” they are often not subject to the pressure of public opinion in their community [8]. In the past 20 years, “extramarital sex” has become a term that touches the sensitive nerves of Chinese people, and it has even been affixed with pretty labels such as “humanity,” “freedom,” and “right” and is regarded as “conspicuous consumption,” reflecting social status, individual charm, and free personality [9].

According to the 2016 Beijing Intermediate People’s Court “White Paper on Specialized Trial Involving Family Disputes,” people born in the 1980s became the main divorced group, and divorces caused by extramarital affairs accounted for 45% of all divorce cases, the first major reason [10]. Extramarital affairs are often accompanied by extramarital sex and significantly affect marriage stability and family integrity. Extramarital sex often hurts and pains the relationship between husband and wife [11] and is a precursor to the breakdown of the marital relationship [12]; in particular, for first-married people, extramarital sex can significantly increase the possibility of divorce and separation of husband and wife [1]. In addition to causing great damage to intimate relationships, extramarital sex can also cause serious psychological, relationship, and health damage to children (including illegitimate children) [13]. Moreover, extramarital sex can lead to the spread of acquired immunodeficiency syndrome (AIDS) and sexually transmitted diseases (STDs), causing serious public health risks; in particular, the number of extramarital sex partners is highly correlated with the risk of contracting STDs [14,15]. Data from a survey in China from 1989 to 1998 showed that the proportion of people infected with sexually transmitted diseases caused by extramarital sex increased year by year, especially from 1995 to 1998, and there was a very significant increase [8]. In addition, social principles and ethical pressure force both parties who have sex outside of marriage to carefully hide their relationship for a long time, causing long-term psychological distress and burden [16].

In China, although the topic of extramarital sex has attracted increasing attention from public opinion, it has not been fully studied in academic circles. Most quantitative studies present descriptive statistics of small samples, and there are few quantitative analyses based on scientific sampling survey methods that are comparable to relevant international studies. The most recently published Chinese General Social Survey 2013 (CGSS2013) incorporates the question of how to consider extramarital sex. This paper uses this question as a dependent variable and draws on relevant literature to explore the influence of different factors (such as education, religion, gender, age, and the Internet) on the acceptance of extramarital sex. Given the nature of the data in the CGSS survey, this study used a zero-inflated Poisson regression model. This paper not only supplements previous research deficiencies but also provides a reference for future study.

## 2. Literature Overview and Study Hypotheses

### 2.1. Literature Overview

The public acceptance of extramarital sex is an important issue in the world today. The 30 year data of the US General Social Surveys from 1991 to 2018 show that the incidence of extramarital sex among people with a history of marriage was 14.63% and 16.48% in 1991 and 2018, respectively [17]. The actual occurrence of extramarital sex is always accompanied by the public’s attitude toward extramarital sex: in 1960–1990, the American people’s tolerance for extramarital sex significantly increased [18]. A panel data study on American society’s sexual behavior and sexual attitudes from 1972 to 2012 noted that Americans’ attitudes toward extramarital sex became increasingly tolerant during this period. The popularity of higher education and the influence of social elites were important factors that contributed to this change [4]. The intergenerational differences in the social attitudes of adults at different birth stages are obvious. Young people born in the 1980s are more tolerant of various controversial issues, such as atheism, homosexuality, and the support of public space for marginal groups [19].

In English literature studies on the acceptance of extramarital sex, individuals’ social background characteristics are found to significantly affect their acceptance with regard to extramarital sex [20]. Religion, education, gender, and social freedom are the core independent variables [21,22,23]. Tolerance of extramarital sex is significantly affected by race (whites are more tolerant than blacks), age (the older a person is, the higher their tolerance), population size of the residential community (the larger the size is, the higher the tolerance), and political orientation (liberals are more tolerant than neutrals and conservatives) [17]. Religion, as a manifestation of social control, has an important impact on sexual fidelity in marriage. The degree of participation in religious activities, the degree of dependence on religious organizations, and the degree of belief in religion are all significantly related to marital fidelity [24]. Historically, with respect to Christian sexuality [21], a study in Germany showed that Christians’ tolerance of extramarital sex was significantly lower than that of other groups [25]. Most studies in other countries support this conclusion [6]. Due to the special status of Christianity in American society, American scholars’ studies have deeply examined different factions of Christianity, piety, and the influence of religious participation. In contrast, conservative sects and more religious groups in Christianity identify with extramarital sex to a lower degree [5,21]. Data analysis shows that groups with more years of education have a higher tolerance toward homosexuality [26]. In a broader social context, some academic studies note that the change in attitudes of European and American societies toward extramarital sex is a manifestation of the “liberalization” and “diversification” of the entire social attitude. In the case of controlling objective conditional variables, individuals’ degree of “liberalization” is positively correlated with their degree of recognition of extramarital sex [4,19].

Regarding marriage, husbands and wives are required to be loyal to each other at both legal and moral levels, and extramarital sex is a manifestation of unfaithful marriage. A survey in the United Kingdom showed that different age groups have very different attitudes toward the principle of sex exclusivity in marriage and toward sex partners outside marriage. Young women have the most open attitude toward extramarital sex, and the waiting time for their first extramarital sexual partner after marriage is shortest; the people attaching the least importance to sexual fidelity are those who have been married for more than ten years and have one or more extramarital sexual partners, while remarried women value sexual fidelity most [27]. A survey in the United States also showed that sexual fidelity is significantly different between married and cohabiting persons [28]. Data of the US General Social Surveys from 1991 to 1996 show that past divorce history, education level, age at first marriage, income, and professional status significantly affect extramarital sex [29].

Relatively speaking, most studies in China are limited to the analysis of specific groups’ recognition of extramarital sex, such as the recurrent population [30] and container truck drivers [31], and quantitative studies involve simple descriptive statistics of small-scale sample data. For example, a small-scale empirical study in Hong Kong showed that young people have a higher acceptance of extramarital sex, and extramarital sex leads to a decrease in marriage satisfaction, which significantly increases the possibility of divorce [9]. Due to the lack of survey data and the neglect of research directions, the sociological community rarely conducts in-depth quantitative analysis of the public acceptance of extramarital sex. Hu [32] noted in his study that the open sexual attitude of the Chinese differs among different provinces. With the Yangtze River as the boundary, the people north of the Yangtze River are more traditional, whereas the people south of the Yangtze River are more open and have a higher level of acceptance of extramarital sex. However, this study neither controlled for other influencing factors nor conducted an in-depth interpretation of individuals’ characteristics. Extramarital sex has been heatedly discussed in public space in China. It is foreseeable that with the opening of public opinion and promotion by prominent social celebrities, this issue will lead to more social attention. The analysis of this important issue can respond to real-life concerns in a timely manner and can be compared with similar studies in other countries, triggering more theoretical thinking.

The most recently published Chinese General Social Survey 2013 (CGSS2013) provides research data to bridge this academic gap. In the CGSS2013, the respondents were asked, “do you think extramarital sex is right?” This paper used the answers to this question to measure the public’s recognition of extramarital sex. The CGSS2013 also surveyed the respondents’ religious beliefs and other sociodemographic characteristics. The inclusion of this question in the CGSS indicated that “sex” is a concern of the public to some extent and is an emphasis in the field of sociological research.

### 2.2. Study Hypotheses

Referring to the above literature, the hypotheses of this study are as follows.

**Hypothesis** **H1.** *The younger the age is, the higher the recognition of extramarital sex.*

American sexual attitude survey results in the 1970s showed that adults with younger ages had a higher level of acceptance of extramarital sex [33]. Recent studies have confirmed this phenomenon. Follow-up studies show that since the 1970s, acceptance of extramarital sex in European and American societies has gradually increased, and the increase in the younger generation’s acceptance has been particularly significant [4,19].

**Hypothesis** **H2.** *An increase in educational level has a positive influence on the acceptance of extramarital sex.*

The positive influence of education on support for extramarital sex is based on a number of reasons. First, education can foster a tolerant mentality; second, education can promote awareness of new things; third, education itself tends to be liberalized [26].

**Hypothesis** **H3.1.** *Christian belief has a negative influence on the acceptance of extramarital sex.*

**Hypothesis** **H3.2.** *Islamic belief has a negative influence on the acceptance of extramarital sex.*

Follow-up studies in Australia show that religion is consistently a key factor influencing the acceptance of extramarital sex; the greater people’s religious participation is, the more negative their attitude toward extramarital sex [34]. The influence of religious factors on the recognition of extramarital sex has been confirmed by Western literature [35]. Both Muslims and Christians have a stronger negative attitude toward extramarital sex [6,36].

**Hypothesis** **H4.** *Internet information has a positive influence on the acceptance of extramarital sex.*

The popularity of the Internet has promoted the destigmatization of extramarital sex [37]. It is not rare for celebrities and stars to admit to extramarital affairs, and reports and discussions about extramarital affairs are ubiquitous in the Internet world. Therefore, groups that are more affected by network information are more likely to be desensitized to extramarital sex.

**Hypothesis** **H5.** *The stronger the tendency of “liberalization” is, the more tolerant people’s attitudes are toward extramarital sex.*

A study that used Chinese college students as survey respondents confirmed that attitudes toward “modern culture” are key factors that influence a group’s sexual cognition [38]. Sexual liberalization is considered by some scholars to be a reflection of the “liberalization” trend of social attitudes throughout Western society [4,19]. Groups with liberal tendencies are often more “tolerant” toward the sexual orientation of others [39].

This paper also discusses the influence of other variables, such as gender and income.

## 3. Data, Variables and Model

### 3.1. Data Source

The study used data from the CGSS2013. The CGSS is China’s first nationwide, general, and continuous large-scale social survey project. Since 2003, it has been conducted annually for individuals in 10,000 households from 125 counties (districts), 500 subdistricts (townships, towns), and 1000 neighborhood committees (village). Through the regular, systematic collection of data on all aspects of China and Chinese society, it summarizes long-term trends in social changes, explores social issues with significant theoretical and practical significance, promotes the openness and sharing of domestic social science research, and provides data and information for international comparative research. The distribution of samples in the CGSS dataset is shown in Table A1 in the Appendix.

CGSS survey data and other survey data are publicly available. These data have a significant influence at home and abroad and are regarded as one of the most important data sources for research on China (See Appendix A. Note nr. 2). The selected variables from the CGSS2013 are indicated below.

### 3.2. Variables

#### 3.2.1. Dependent Variables

“Do you think extramarital sex is right?” The possible answers were 1. always wrong; 2. wrong in most cases; 3. hard to say whether it is right or wrong; 4. sometimes right; and 5. completely right. As shown in the simple descriptive statistical findings (see Table 1), among the answers to this question, respondents who chose the option “always wrong” accounted for the absolute majority, approximately 80%. It is clearly indicated that the proportion accepting extramarital sex is only 2.33% (1.74 + 0.59), which is much smaller. Due to the special distribution of the answers, the classic Poisson distribution and negative binomial distribution are not suitable. This study used a zero-inflated model. Zero-inflated Poisson regression models have been widely used in research fields such as medicine, finance, insurance, and sociology [40]. The authors recoded the data as 0, 1, 2, 3, and 4 (e.g., 0 means “always wrong”).

#### 3.2.2. Independent Variables

1. Age group (A3) (See Appendix A. Note nr. 3): According to the respondents’ answers, their ages were recoded into different age groups according to their date of birth: born in the 1990s, 1980s, 1970s, 1960s, 1950s, and 1940s and before.
2. The highest level of education (A7a): According to the answers, the answers were recoded as primary school and below, junior high school, senior high school (technical secondary school/technical school), junior college education, undergraduate education, and postgraduate education and above.
3. Gender (A2): Dichotomous variable: male and female.
4. Income (A62): Family income after taking the logarithm.
5. Religious belief (A5): This paper selected four dichotomous variables, namely, Buddhist, Taoist, Muslim. and Christian beliefs, and coded Catholic, Christian, Orthodox, and other Christian beliefs into a dichotomous variable. In response to the question “do you believe in Christianity?”, 1 means yes, and 0 means no.
6. Whether the Internet is the main source of information (A29): According to the respondents’ answers, the answers were recoded into one dichotomous variable: the Internet is the main source of information, and media other than the Internet (television, newspapers, etc.) are the main source of information.
7. Social attitude variable: 1. (A46) If someone criticizes the government in a public place, the government should not interfere. 2. (A47) The number of children you want to have is a personal matter, and the government should not interfere. Do you agree? The options are 1. strongly disagree, 2. disagree, 3. uncertain, 4. agree, and 5. strongly agree.

#### 3.2.3. Study Model

The study used a zero-inflated Poisson regression model. In the real world, it is often found that the number of occurrences of the research objects (count variable) contains a large proportion of zero values, such as insurance claims, students dropping out of school, the number of divorces, and the number of induced abortions. Due to their own distribution problems, such data exceed the analysis capabilities of general count models such as Poisson and negative binomial models. A severely high probability of a zero point in the investigation and survey will lead to excessive dispersion (i.e., the zero inflation problem) [40,41].

The model considers the zeros in the count data as “structural zeros” (extra zeros) and “sampling zeros” (true zeros), conducts segmentation from zero, establishes a mixed probability distribution for zero counts and nonzero counts, and establishes a logit model and a general count (Poisson) model for the zero part and the nonzero part, respectively, to address the problem of excessive zeros in the data.

In the zero-inflated model, zeros are generated by two different parts. The probability that the first part generates zeros is called structural zeros or extra zeros, and the zeros of the second part are generated by a model obeying a discrete distribution, Poisson distribution, and so on, called sampling zeros. In the model, the distribution function of Y is as follows:

(1) $$\begin{array}{l} \mathrm{Pr}\left(y_{j}=0\right)=\pi +\left(1-\pi \right)e^{-\lambda }\\ \mathrm{Pr}\left(y_{j}=h_{i}\right)=\left(1-\pi \right)\frac{\lambda^{h_{i}}e^{-\lambda }}{h_{i}!},\;h_{i}\geq 1 \end{array}$$

π indicates the probability of extra zeros, and Y in the second part obeys the discrete Poisson distribution. Correspondingly, the zero-inflated Poisson regression is divided into two processes for calculation [42]. The process obeying the Poisson distribution uses general Poisson regression, and the interpretation method is similar to Poisson regression. For the zero-inflated part (logit model), the influence of a particular variable on the probability that the dependent variables take a structural zero is examined. If the coefficient of the part is negative, the increase in the coefficient will reduce the probability that the dependent variables take a zero.

## 4. Data Analysis

### 4.1. Descriptive Statistical Results of Independent Variables

As shown in Table 2, in the nationwide samples drawn by the CGSS2013, the number of male respondents was basically the same as that of female respondents, accounting for 50% each. The age groups of the respondents were separated by 10 years. For those born in the 1940s to the 1970s, each age group accounted for approximately 20% of the total respondents, those born in the 1980s accounted for 14.9%, and those born in the 1990s accounted for a small proportion, only 6.1%. Thirteen percent of the respondents had not received any education, and the number of respondents with junior high school education was largest, accounting for nearly 30%. Respondents with a junior college education or above accounted for 16.3%. The proportions of the respondents who believed in Buddhism, Islam, and Christianity were 5.1%, 2.1%, and 2.0%, respectively. More than two-fifths (43.5%) of the respondents believed that the government should not interfere with public criticism. More than two-thirds (61.2%) of the respondents believed that the government should not interfere with childbirth. Although the Internet has become more popular in China, the number of respondents who used the Internet as the main source of information was not high, accounting for only 20%.

### 4.2. Results of Regression Analysis

In the statistical application, the advantages and disadvantages of the zero-inflated model and the ordinary Poisson regression model were compared through the Voong value test. The Voong value test of all the models in this paper was *p* < 0.01, indicating that the zero-inflated Poisson regression model is superior to the ordinary model (see Table 3).

Model 1 incorporated three basic variables, gender, age, and income. The results of the model showed that gender had no significant influence on the degree of acceptance of extramarital sex. The influence of age was significant, and the regression coefficient ranged from 0.244 to 0.748 according to the results of the comparison of those born in the 1950s–1990s and those born in the 1940s, indicating that the younger the people were, the higher their level of acceptance of extramarital sex. An increase in income had a positive influence on the acceptance of extramarital sex.

Based on Model 1, Model 2 incorporated the education variable. After controlling for the education variable, the influence of income became less significant. For people with different levels of education, there were significant differences in their acceptance of extramarital sex. The higher the level of education was, the more people that accepted extramarital sex. In the case of controlling other variables, the influence of age on extramarital sex was still significant. Model 3 incorporated the religion variable. In the case of controlling for age, education, and other variables, there was no significant difference with respect to the acceptance of extramarital sex for Buddhists and Christians. However, the influence of Islamic belief was significant, and the groups that believed in Islam had a lower level of acceptance of extramarital sex. Model 4 incorporated the social attitude variable. Referring to the relevant literature, two questions in the CGSS were used to measure the freedom tendencies of the respondents in this study. The results showed that the groups with higher freedom tendencies had a higher level of acceptance of extramarital sex. After controlling for social attitude, the difference between the groups born in the 1980s and 1990s and other groups remained significant. Highly educated groups had a higher level of acceptance of extramarital sex. Model 5 incorporated the Internet variable, and the selected variable was “whether the Internet is the main source of information.” The more frequently respondents used the Internet, the more likely the Internet was to be their main source of information, and the higher their level of acceptance of extramarital sex.

By summing the results of the above models, the Chinese survey data show that the factors that significantly influence the tolerance of extramarital sex include age, education, Islamic belief, social attitude, and Internet use. However, factors such as gender, Buddhist and Christian beliefs, and income have no significant influence on the tolerance of extramarital sex.

**Figure 1:**
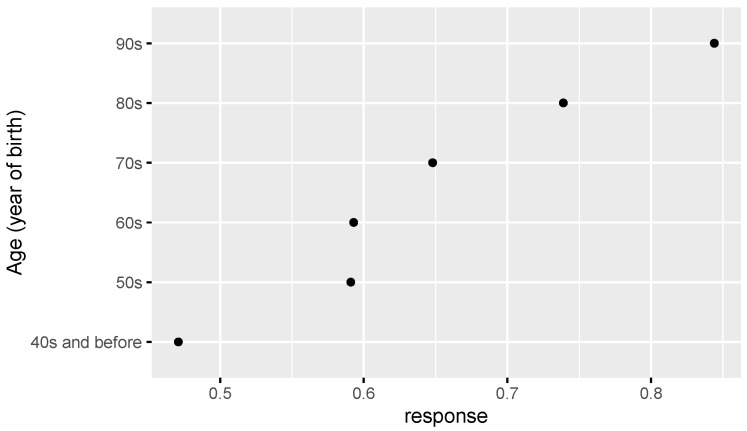
Zero-inflated regression coefficient of age.

**Figure 2:**
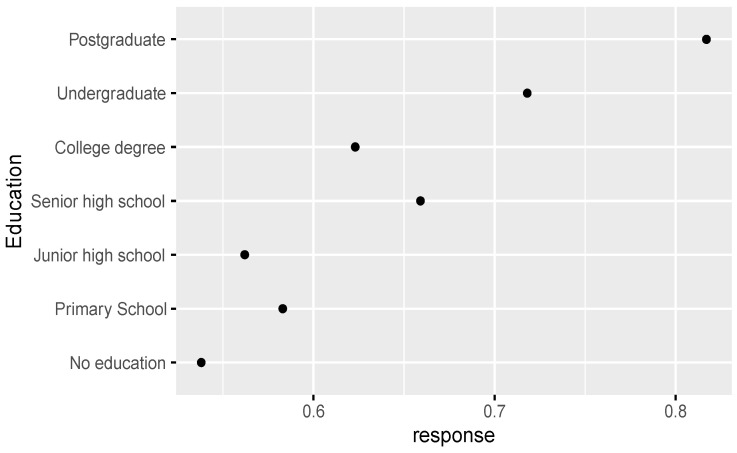
Zero-inflated regression coefficient of education.

**Figure 3:**
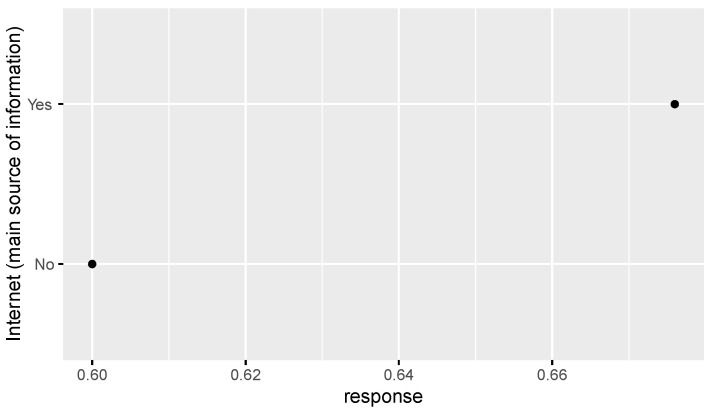
Zero-inflated regression coefficient of Internet.

**Figure 4:**
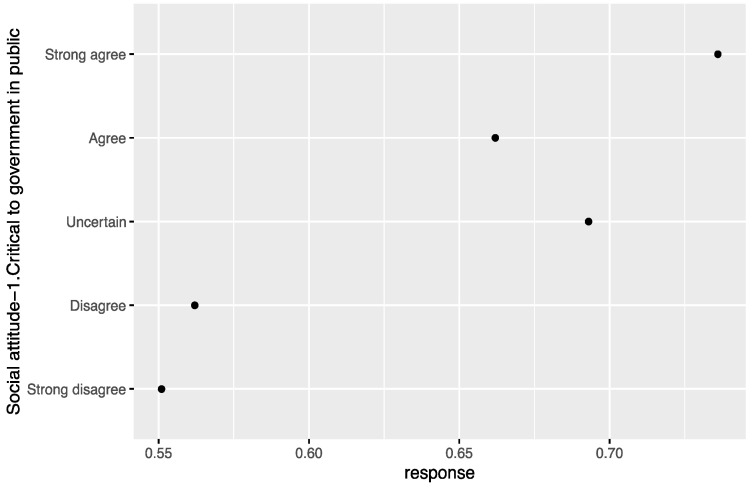
Zero-inflated regression coefficient of social attitude-1.

**Figure 5:**
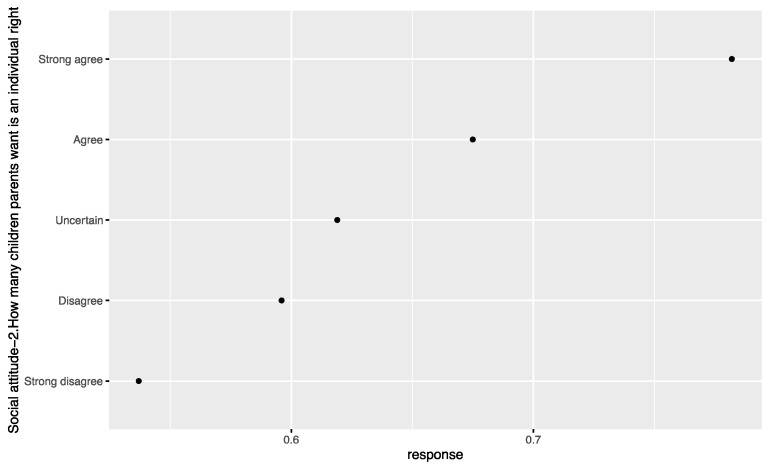
Zero-inflated regression coefficient of social attitude-2.

**Figure 6:**
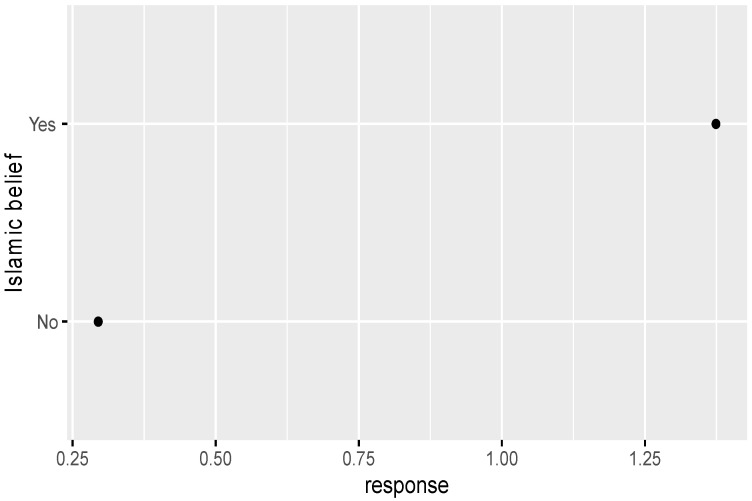
Zero-inflated regression coefficient of Islamic belief.

Based on the comparative analysis of zero-inflated regression coefficients for different levels of factors such as age, education, Islamic belief, social attitude, and Internet use that have a significant influence on tolerance of extramarital sex, Figure 1 shows that the regression coefficient of those born in the 1980s-1990s is significantly higher than that of other age groups, and those born in the 1950s–1970s have a similar level of acceptance of extramarital sex. Those with an undergraduate education or above have an obviously higher level of acceptance of extramarital sex than those with a junior college education or below (see Figure 2). People who rely on the Internet as their source of information (see Figure 3) and whose social attitudes tend to be more open and liberal (see Figure 4 and Figure 5) have a higher level of acceptance of extramarital sex. Note that non-Muslims have an obviously higher level of acceptance of extramarital sex than Muslims do. Belief in Islam has an obviously stronger influence on the acceptance of extramarital sex than other factors do (see Figure 6).

## 5. Conclusions and Discussion

### 5.1. Conclusions

Study hypothesis 1 (age) was verified. The age groups born in the 1980s and 1990s are an obvious division, and their regression coefficients are significantly higher than those of other age groups. We can expect that the younger generation will be more open to extramarital sex. Study hypothesis 2 (education) was verified, and the analysis shows that good higher education is a key distinction. The higher the education level was, the greater the acceptance of extramarital sex. In particular, people who have received undergraduate education or above have a higher level of acceptance of extramarital sex. Study hypothesis 3.1 (Christianity) was not supported. Unlike the evidence in other countries, the Chinese survey data do not support the significant influence of Christian belief on the acceptance of extramarital sex. In this regard, more detailed investigations and studies are needed. A possible reason is that Chinese Christians are mainly Protestants rather than traditional Catholics, and thus, they have greater freedom for personal sexual behavior and loyalty to marriage. Study hypothesis 3.2 (Islam) was verified. Islamic belief has a negative influence on the acceptance of extramarital sex. Study hypothesis 4 (the Internet) was verified to some extent. The probability that the group that uses the Internet as their main source of information is completely opposed to extramarital sex is lower than that of the reference group. Study hypothesis 5 (liberalization) was verified. On average, the stronger the respondents’ support for personal choice and freedom rights was, the greater their acceptance of extramarital sex. To some extent, desensitization to extramarital affairs in Chinese society in recent years is a manifestation of the overall trend of society toward freedom of thought and pluralism. The Chinese data do not support a significant influence of gender on the acceptance of extramarital sex. Men and women have relatively consistent views and attitudes toward extramarital sex, and there is no gender difference. After controlling for variables such as education, income has no significant influence.

### 5.2. Discussion

Academic studies on issues related to extramarital sex in China lag behind social development. Although the government has not issued policies on extramarital sex, the public’s discussion of extramarital sex has been increasing. The media’s discussion of extramarital affairs is more common, and the expression of extramarital sex groups’ own appeals has gradually entered the public space. Marriage is based on the feelings of the spouses, and individuals may perform extramarital sex for various reasons, such as excitement seeking, impulsiveness, new experiences, satisfaction, or spiritual emptiness [12]. However, considering traditional marriage and family views in China, mainstream society is still committed to maintaining the stability and integrity of families. Marriage and family are still very important in social culture. Existing studies show that even if parties have had extramarital sex, they still value marriage and family life and try their best to manage marriage and raise children [18]. The occurrence of extramarital sex does not necessarily mean that the spouses’ emotions have already declined and that marriage is irreparable. From the perspective of social morality and ethics, effectively intervening or even reducing the public’s acceptance of extramarital sex can prevent the actual risk of extramarital sex to a certain extent and maintain the overall stability of family life in society. This presents a wide space for family services to provide marriage and family counseling and assist clients in making rational choices.

This study shows that people of younger ages, higher educational levels, stronger tendencies toward liberalization, and greater dependences on Internet information have a higher degree of acceptance of extramarital sex. Although extramarital sex is a personal behavior, it is always contrary to modern marriage and family views. It is necessary to provide marriage and family education for young people and guide young people to understand the relationship between freedom and self-respect, analyze Internet information, and cultivate correct marriage and family views. Therefore, family counseling services need to enhance the connection between individuals and mainstream society and increase their attachment to society through corresponding group or community activities to prevent the emergence of extramarital sex and marital problems.

In China, there is no correlation between gender and acceptance of extramarital sex. Note that the acceptance of extramarital sex and the actual occurrence are two completely different concepts. Although there is a correlation between cognition and behavior, there is also a huge gap between them. Studies in the United States and the United Kingdom are based on the actual occurrence of extramarital sex as a measure [17,27,29]. The actual incidence of extramarital sex is much higher in men than in women. However, at the cognitive level, this study shows that men and women actually have the same acceptance of extramarital sex. Why does the same acceptance make a difference in actual behavior? This may be related to the reasons why men and women have extramarital sex and actual social tolerance. In extramarital sex, men value sex more and have more sexual curiosity, while women value emotions more and hope to find the “right” person [43,44]; therefore, men are more likely to have extramarital sex. At the same time, men are sometimes regarded as “successful” and “attractive” if they have extramarital sex, while women are labeled as “lewd” and “unchaste” if they have extramarital sex. Society’s moral tolerance for men’s extramarital sex is significantly higher than that of women’s extramarital sex. China has been promoting and practicing gender equality, and men and women have the same acceptance of extramarital sex in the same social environment; however, differences in social tolerance may ultimately affect the actual occurrence of extramarital sex.

If extramarital sex is regarded as a deviant behavior, religion should play a certain role in social control over the deviant behavior [45]. The influence of religious factors differs between China and foreign countries. Relative to other countries, the importance of religious life for the Chinese is very low. According to the survey results of the Pew Research Center, less than 10% of Chinese people believe that religion plays an important role in their lives (See Appendix A. Note nr. 4). Correspondingly, the influence of Christian factors highlighted in the foreign literature on the study of extramarital affairs has not been verified by data. Other religions, such as Buddhism and Taoism, also have no significant influence. In contrast, belief in Islam has a significant influence on the level of acceptance of extramarital sex. With respect to the difference between China and foreign countries for the influence of Christian factors, a possible explanation is that Chinese Christians may be more influenced by the values of European and American countries; thus, they have a relatively higher level of tolerance toward extramarital sex. This factor makes the difference displayed by the internal data in European and American countries insignificant in China.

It is necessary to further think why different religions in China have different effects on people’s acceptance of extramarital sex. Perhaps what we should consider more is the actual degree of participation in religious activities. Foreign studies show that the reduction in married people’s marital infidelity is closely related to the degree of religious devotion and conviction, especially the degree of participation in religious activities, but has nothing to do with beliefs, doctrines, and other religious characteristics [46]; more specifically, the degree of participation in religious activities and the degree of attachment to religious organizations are significantly related to marital fidelity [24]. Christians, Buddhists, and Taoists in China are relatively loosely attached to religious organizations, and they do not frequently participate in religious activities. Most of them choose to participate in religious activities based on their own utilitarian needs: Buddhists and Taoists (except monks) are not required to regularly participate in religious activities, and Christians rarely go to church regularly every week [47]. In comparison, Muslims participate in religious activities more frequently, have to pray several times a day, and are more dependent on religious organizations. For religious reasons, Muslims are more socially controlled, and their attitudes toward extramarital sex are becoming more conservative. As both religion and extramarital affairs are topics that have not received much empirical attention in Chinese social science studies, more in-depth studies are needed on this issue. Furthermore, there are few comparative studies on the acceptance of extramarital sex by different religious groups in Western countries. The findings and interpretation of this study need further cross-cultural verification.

When more European and American countries support extramarital affairs, it can be predicted that various disputes related to extramarital affairs will attract more attention. In English academic circles, studies on extramarital affairs have become more specialized and refined, and there are numerous specialized academic journals studying the issue of extramarital affairs. Chinese academics’ attention to this issue is still in its infancy. This study is of great significance to the study of extramarital affairs, both academically and practically.

## Figures and Tables

**Table 1:** Distribution of attitudes toward extramarital sex.

Attitude toward Extramarital Sex	Freq. ^a^	Percent	Cum. ^b^
Always wrong	8799	78.53	78.53
Wrong in most cases	711	6.35	84.88
Hard to say whether it is right or wrong	1433	12.79	97.67
Sometimes right	195	1.74	99.41
Completely right	66	0.59	100.00
Total	11,204	100.00	

Notes: a. Freq. = Frequency. b. Cum. = Cumulative.

**Table 2:** Descriptive statistics of variables.

Variable	Freq	%
Gender		
Male	5756	50.3
Female	5682	49.7
Date of birth		
Born in the 1940s and before	2269	19.8
Born in the 1950s	2223	19.4
Born in the 1960s	2300	20.1
Born in the 1970s	2252	19.7
Born in the 1980s	1700	14.9
Born in the 1990s	693	6.1
The highest level of education		
No education	1484	13.0
Primary school education	2582	22.6
Junior high school education	3326	29.1
Senior high school/technical secondary school education	2180	19.1
Junior college education	927	8.1
Undergraduate education	843	7.4
Postgraduate education and above	90	0.8
Your religious belief—Buddhism		
No	10,849	94.9
Yes	584	5.1
Your religious belief—Islam		
No	11,192	97.9
Yes	241	2.1
Your religious belief—Christianity		
No	11,204	98.0
Yes	234	2.0
If someone criticizes the government in a public place, the government should not interfere		
Strongly disagree	1004	8.9
Disagree	3915	34.6
Uncertain	3012	26.6
Agree	2737	24.2
Strongly agree	646	5.7
The number of children you want to have is a personal matter, and the government should not interfere		
Strongly disagree	1990	17.5
Disagree	4984	43.7
Uncertain	1782	15.6
Agree	2206	19.4
Strongly agree	436	3.8
Whether the Internet is the main source of information		
The Internet is not the main source of information	8884	80.0
The Internet is the main source of information	2224	20.0

**Table 3:** Results of zero-inflated Poisson regression analysis.

Independent Variable ^a^		Dependent Variable: Do You Think Extramarital Sex Is Right?				
		Model 1	Model 2	Model 3	Model 4	Model 5
Gender (reference group: male)						
Female	Regression coefficient *b*	−0.00246	−0.00964	−0.0120	−0.0274	−0.0289
	Standard deviation	(0.0391)	(0.0395)	(0.0395)	(0.0396)	(0.0400)
	Incidence ratio *e^b^*	0.998	0.990	0.988	0.973	0.971
Age (reference group: born in the 1940s and before)						
Born in the 1950s		0.244 **	0.226 **	0.235 **	0.228 **	0.250 **
		(0.0805)	(0.0817)	(0.0815)	(0.0814)	(0.0831)
		1.277	1.254	1.265	1.256	1.284
Born in the 1960s		0.281 ***	0.248 **	0.262 **	0.235 **	0.262 **
		(0.0794)	(0.0826)	(0.0824)	(0.0823)	(0.0838)
		1.325	1.282	1.300	1.265	1.299
Born in the 1970s		0.399 ***	0.353 ***	0.363 ***	0.338 ***	0.347 ***
		(0.0761)	(0.0808)	(0.0806)	(0.0808)	(0.0830)
		1.490	1.424	1.437	1.403	1.415
Born in the 1980s		0.610 ***	0.496 ***	0.504 ***	0.490 ***	0.475 ***
		(0.0744)	(0.0818)	(0.0815)	(0.0821)	(0.0861)
		1.840	1.642	1.655	1.632	1.608
Born in the 1990s		0.748 ***	0.635 ***	0.651 ***	0.635 ***	0.605 ***
		(0.0864)	(0.0941)	(0.0939)	(0.0945)	(0.0993)
		2.113	1.888	1.917	1.887	1.832
Income		0.0120 ***	0.00608	0.00608	0.00373	0.00364
		(0.00349)	(0.00392)	(0.00395)	(0.00401)	(0.00404)
		1.012	1.006	1.006	1.004	1.004
Level of education (reference group: no education)						
Primary school education			0.0527	0.0479	0.0751	0.0671
			(0.0912)	(0.0907)	(0.0903)	(0.0933)
			1.054	1.049	1.078	1.069
Junior high school education			0.0178	0.00946	0.0449	0.0352
			(0.0901)	(0.0896)	(0.0898)	(0.0928)
			1.018	1.010	1.046	1.036
Senior high school/technical secondary school education			0.193 *	0.181 *	0.224 *	0.195 *
			(0.0918)	(0.0913)	(0.0913)	(0.0949)
			1.213	1.198	1.251	1.216
Junior college education			0.157	0.149	0.191	0.154
			(0.103)	(0.103)	(0.103)	(0.107)
			1.170	1.160	1.210	1.166
Undergraduate education			0.320 **	0.308 **	0.344 ***	0.288 **
			(0.0999)	(0.0998)	(0.100)	(0.106)
			1.377	1.360	1.410	1.333
Postgraduate education and above			0.504 ***	0.500 ***	0.483 ***	0.414 **
			(0.146)	(0.146)	(0.146)	(0.151)
			1.656	1.649	1.621	1.513
Religious belief						
Buddhist belief (reference group: without Buddhist belief)				0.0910	0.114	0.101
				(0.0859)	(0.0865)	(0.0878)
				1.095	1.121	1.107
Islamic belief (reference group: without Islamic belief)				−1.616 ***	−1.544 ***	−1.516 ***
				(0.291)	(0.290)	(0.290)
				0.199	0.214	0.220
Christian belief (reference group: without Christian belief)				−0.0753	−0.0367	−0.0482
				(0.147)	(0.147)	(0.151)
				0.927	0.964	0.953
Public criticism of the government is personal freedom, and the government should not interfere (reference group: strongly disagree)						
Disagree					0.0350	0.0199
					(0.0960)	(0.0970)
					1.036	1.020
Uncertain					0.243 *	0.226 *
					(0.0980)	(0.0990)
					1.275	1.254
Agree					0.198 *	0.184
					(0.0969)	(0.0978)
					1.218	1.202
Strongly agree					0.312 **	0.293 **
					(0.112)	(0.113)
					1.366	1.340
The number of children you want to have is personal freedom, and the government should not interfere (reference group: strongly disagree)						
Disagree					0.0966	0.118
					(0.0662)	(0.0668)
					1.101	1.125
Uncertain					0.133	0.154
					(0.0788)	(0.0795)
					1.142	1.167
Agree					0.220 **	0.235 **
					(0.0721)	(0.0728)
					1.246	1.265
Strongly agree					0.375 ***	0.380 ***
					(0.0970)	(0.0983)
					1.455	1.462
Whether the Internet is the main source of information (reference group: other media are the main source of information)						0.111 *
						(0.0542)
						1.118
Observations		10042	10038	10034	9937	9674
Chi-square		125.805 ***	157.260 ***	196.891 ***	256.513 ***	441.777 ***
Nag R-square		0.016	0.020	0.025	0.034	0.058

(See Appendix A. Note nr. 1) Notes: * *p* < 0.05, ** *p* < 0.01, *** *p* < 0.001. a. The regression coefficient b (standard deviation) and incidence ratio *e*^b^ are given with respect to the regression results of the variables.

## Data Availability

The data of this study are available from the authors upon request.

## Appendix A. Notes

1. Through the government’s purchase of social services, these centers will be operated by social service agencies. One comprehensive family service center requires 20 full-time staff members, including at least 14 professional social workers.
2. See www.chinagss.org/, (accessed on 1 March 2021)
3. The title number here is the number of survey questions in the CGSS questionnaire for easy reference.
4. See http://www.pewresearch.org/fact-tank/2015/12/23/americans-are-in-the-middle-of-the-pack-globally-when-it-comes-to-importance-of-religion/, (accessed on 25 February 2021)

**Table A1:** Distribution of CGSS data in different provinces of China.

Province	Household Registration		
	Urban Hukou	Rural Hukou	Total
Beijing	594	0	594
Tianjin	432	0	432
Hebei	100	201	301
Shanxi	200	100	300
Inner Mongolia	25	75	100
Liaoning	353	50	403
Jilin	227	278	505
Heilongjiang	325	277	602
Shanghai	558	0	558
Jiangsu	379	125	504
Zhejiang	371	123	494
Anhui	151	251	402
Fujian	200	100	300
Jiangxi	301	200	501
Shandong	325	275	600
Henan	225	375	600
Hubei	350	252	602
Hunan	275	225	500
Guangdong	378	0	378
Guangxi	201	200	401
